# Synthesis and activity of three new trinuclear platinums with *cis*-geometry for terminal metal centres

**DOI:** 10.1186/1423-0127-21-41

**Published:** 2014-05-12

**Authors:** Shahnaz A Hamad, Philip Beale, Jun Q Yu, Fazlul Huq

**Affiliations:** 1 The University of Sydney, Cumberland Campus, Lidcombe, Sydney, NSW 2141, Australia; 2 Sydney Cancer Centre, Concord Hospital, Sydney NSW 2139, Australia

**Keywords:** Ovarian cancer, Platinum drug, Trinuclear, A2780, pBR322 plasmid, Drug resistance, Pt−DNA binding

## Abstract

**Background:**

As compared to cisplatin, trinuclear platinum compounds such as BBR3464 and DH6Cl have an altered spectrum of activity possibly because they form long-range adducts with DNA as against mainly intrastrand 1,2-bifunctional adducts formed by cisplatin and its analogues. Because of the labilizing effect associated with the *trans*-geometry, the compounds are expected to break down inside the cell thus serving to reduce the number of long-range adducts formed. In contrast, trinuclear platinum complexes with *cis*-geometry for the terminal metal centres would be less subject to such breakdown and hence may produce a greater number of long-range inter- and intrastrand adducts with the DNA. This paper describes the synthesis and activity against human ovarian tumour models of of three new trinuclear platinum complexes with *cis*-geometry for terminal platinum centres, coded as QH4, QH7 and QH8. The paper also describes cellular accumulation of platinum, level of drug−DNA binding, and nature of interaction of the compounds with pBR322 plasmid DNA.

**Results:**

Methods of synthesis, elemental analysis, spectral studies and molar conductivity measurements provide support to the suggested structures of the compounds. QH4 and QH8 are found to be more cytotoxic than cisplatin against the parental A2780 cell line; QH8 is more active than cisplatin against the resistant A2780^cisR^ and A2780^ZD0473R^ cell lines as well. The least compound QH7 shows a greater activity against the resistant cell lines than the parental cell line; it is most damaging to pBR322 plasmid DNA and most able to induce changes in DNA conformation. The variations in activity of the compounds, changes in intracellular drug accumulation and levels of Pt−DNA binding with the changes in number of planaramine ligands bound to central platinum and the length of the linking diamines, can be seen (1) to illustrate structure-activity relationships and (2) to highlight that the relationship between antitumour activity and interaction with cellular platinophiles including DNA can be quite complex as the cell death is carried out by downstream processes in the cell cycle where many proteins are involved.

**Conclusion:**

Among the three designed trinuclear platinum complexes with *cis-*geometry for the terminal metal centres, the most active compound QH8 is found to be more active than cisplatin against the parental A2780 and the resistant A2780^cisR^ and A2780^ZD0473R^ cell lines.

## Background

**Figure 1:**
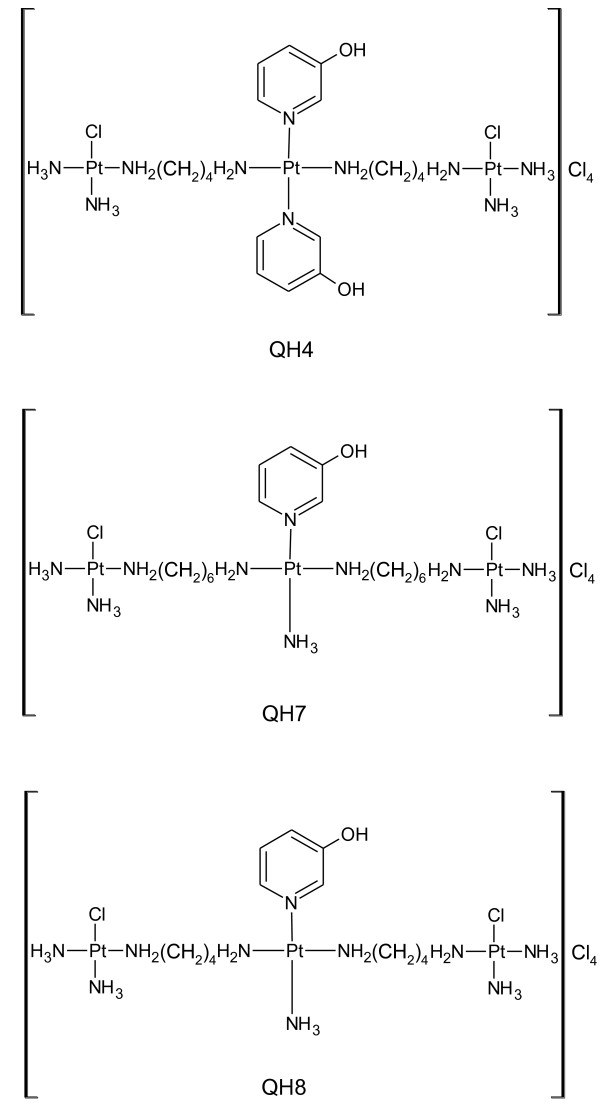
**Structures of QH4, QH7 and QH8.** QH2: [{*cis*-PtCl(NH_3_)_2_}_2_ μ{*trans*-Pt(3-hydroxypyridine)_2_(H_2_N(CH_2_)_4_NH_2_)_2_}]Cl_4_; QH3: [{*cis*-PtCl(NH_3_)_2_}_2_ μ{*trans*-Pt(3-hydroxypyridine)(NH_3_)(H_2_N(CH_2_)_6_NH_2_)_2_}]Cl_4_; QH4: [{*cis*-PtCl(NH_3_)_2_}_2_ μ{*trans*-Pt(3-hydroxypyridine)(NH_3_)(H_2_N(CH_2_)_4_NH_2_)_2_}]Cl_4._

Although cisplatin is a widely used anticancer drug [1,2], its use is also limited due to intrinsic and/or acquired resistance and the presence of numerous side effects [3,4]. Trinuclear platinum compounds such as BBR3464, DH6Cl, DH7Cl, TH1 and CH25 that bind with DNA differently than cisplatin (in the sense that they form long-range adducts with DNA as against mainly 1,2-bifunctional adducts formed by cisplatin) are found to be significantly more cytotoxic than cisplatin [5-9]. Because of the *trans*-geometry for the terminal metal centres, the compounds are expected to break down inside the cell, thus reducing the number of long-range adducts with DNA. The degradation products are expected to bind with cellular thiols such as glutathione that plays key roles in detoxification of reactive oxygen and reactive nitrogen species. In contrast, trinuclear platinum complexes with *cis*-geometry for the terminal metal centres would be less subjected to such breakdown and therefore may produce a greater number of long-range inter- and intrastrand adducts with DNA. The present paper deals with the synthesis, characterization and activity of three new trinuclear platinum complexes with *cis*-geometry for terminal platinum centres, [{*cis*-PtCl(NH_3_)_2_ μ-{*trans-*Pt(3-hydroxypyridine)_2_(H_2_N(CH_2_)_4_NH_2_)_2_}]Cl_4_ (coded as QH4), [{*cis*-PtCl(NH_3_)_2_}_2_ μ-{*trans*-Pt(3-hydroxypyridine)(NH_3_)(H_2_N(CH_2_)_6_NH_2_)_2_}]Cl_4_ coded as QH7 and [{*cis*-PtCl(NH_3_)_2_}_2_ μ-{*trans*-Pt(3-hydroxypyridine)(NH_3_)(H_2_N(CH_2_)_4_NH_2_)_2_}] coded as QH8 (Figure 1). Whereas the two terminal platinum ions bind covalently with DNA, the central platinum ion can only undergo non-covalent interactions including electrostatic interaction and hydrogen bonding via 3-hydroxypyridine ligand.

## Methods

### Materials

3-hydroxypyridine, N,N-dimethylformamide [DMF], dimethyl sulfoxide (DMSO), 1,6-diaminohexane dihydrochloride and putrescine (tetramethylene diamine) (Sigma Chemical Company St. Louise USA); potassium tetrachloroplatinate(II) (K_2_[PtCl_4_]), restriction enzyme (BamH1), 10X digestion buffer and Polaroid black-and-white print film type 667, trypsin, hepes, dulbecco^,^s phosphate buffered saline (PBS) powder, 3-[4,5-dimethylthiazol-2yl]-diphenyl tetrazoliume bromide (MTT) and dimethyl sulfoxide (DMSO) (Aldrich Pty Ltd NSW, Australia); dihydrochloride, sodium hydroxide, silver nitrate (AgNO_3_), potassium chloride (KCl), potassium iodide (KI) ethanol, methanol, acetone, diethyl ether and concentrated hydrochloric acid (HCl) (Alax chemicals Auburn NSW Australia); concentrated ammonia solution, triethyl amine, dichloromethane and 28% ammonia solution (Asia Pacific Speciality Chemicals Ltd Auckland New Zealand); agarose and pBR322 plasmid DNA (ICN Biomedicals Ohio USA); trizma-HCl, trizma base disodium salt of ethylene diamine tetraacetic acid, boric acid, acetic acid and ethidium bromide (Sigma USA); fetal calf serum, 5X RPMI 1640, 200 mM L-glutamine and 5.6% sodium bicarbonate (Trace Bioscience Pty Ltd Australia); commercially available JETQUICK Blood DNA Spin Kit/50 (Astral Scientific Australia).

### Synthesis

**Figure 2:**
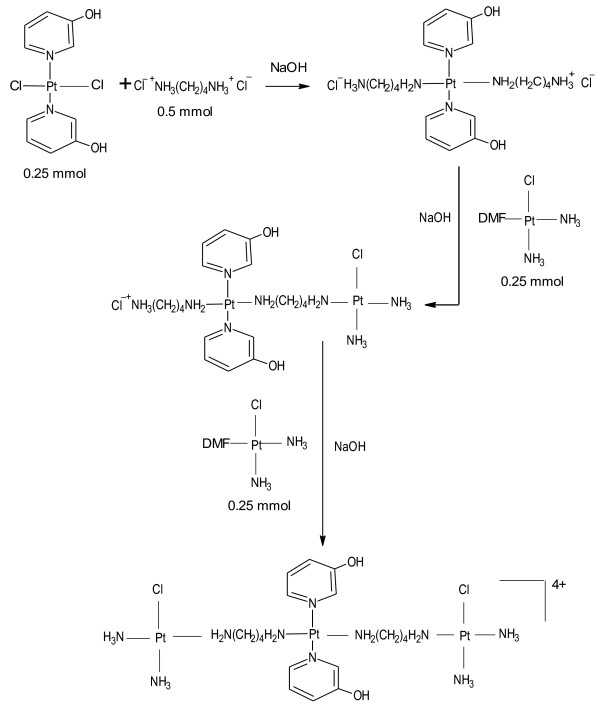
Schema for the synthesis of QH4.

**Figure 3:**
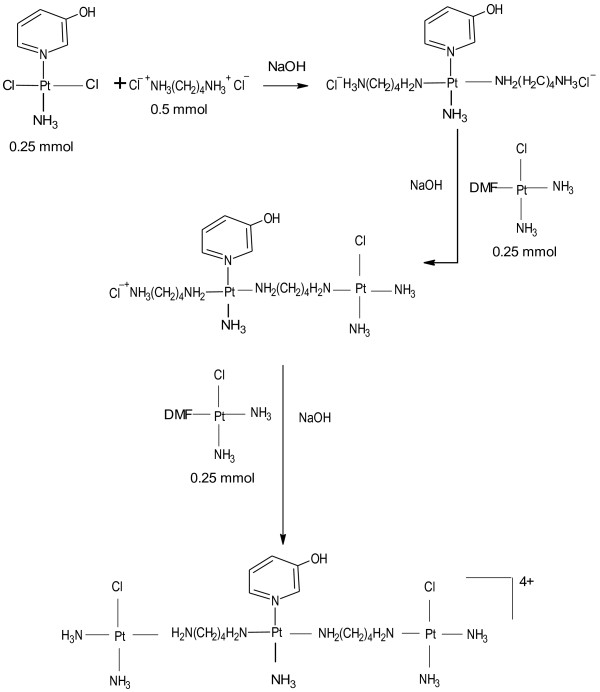
Schema for the synthesis of QH7.

**Figure 4:**
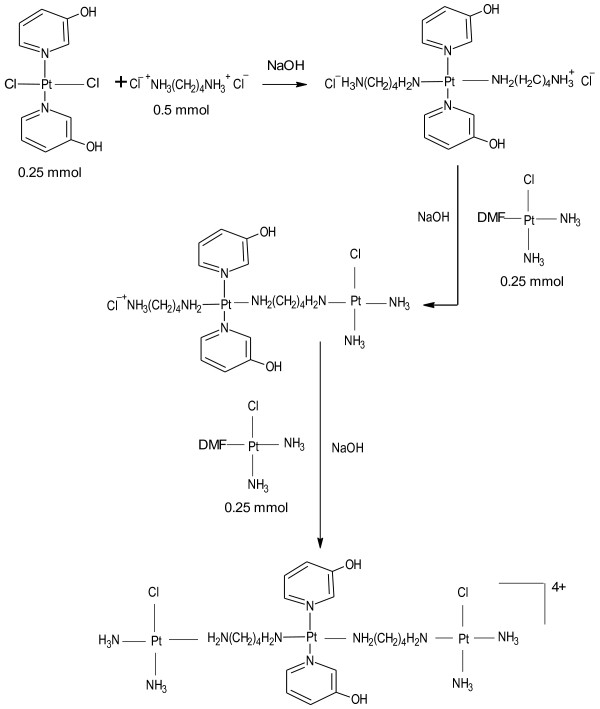
Schema for the synthesis of QH8.

CH1denoting [*trans*-PtCl_2_(3-hydroxypyridine)_2_ required for the synthesis of QH4 and YH11denoting [*trans*-PtCl_2_(NH_3_)(3-hydroxypyridine)] required for the synthesis of QH7 and QH8 were prepared according to previously published method [10]. QH7 and QH8 were prepared following a method similar to that used for the synthesis of TH1 [7]. The method used for synthesis of QH4 was essentially same as that used for the synthesis of QH8 except that CH1 was used instead of YH11. The schemata for synthesis of QH4, QH7 and QH8 are given in Figures 2, 3 and 4 respectively.

Briefly, cisplatin (0.5 mmol, 0.15 g) was dissolved in 10 mL of DMF to which was added 0.495 mmol of silver nitrate (0.0849 g). The mixture was stirred at room temperature for 24 h in the dark and then centrifuged (at 4800 rpm for 30 min, 21°C) to remove precipitate of AgCl. The supernatant containing a solution of *cis*-Pt(NH_3_)_2_Cl(DMF) was collected and kept at −16ºC. A suspension of 0.25 mmol (0.0945 g) of YH11 or 0.25 mmol (0.114 g) of CH1 in 10 mL of DMF was gently heated with stirring at 60°C for 20 min. In the case of QH4 and QH8 0.5 mmol of 1, 4-diaminobutane dihydrochloride (0.085 g) was dissolved in 4 mL of DMF and in the case of QH7 0.5 mmol of 1, 6-diaminohexane (0.058 g) was dissolved in 4 mL of DMF to which 1 mL of 1 M HCl was added dropwise with stirring. The diamine solution was stirred for a further 15 min. It was then added to YH11 suspension dropwise with stirring within 30 min of its preparation followed by the addition of 0.5 mL of 1 M NaOH with stirring at 70°C. Stirring was continued at 60°C for about 1 h, then for 4 h at 50°C followed by the addition of 200 μL of 1 M NaOH. The mixture was stirred for further 5 min at 50°C to which was added cisplatin supernatant (0.25 mmol) with stirring. The temperature of the mixture was increased to 70°C and stirred for a further 5 min. A further 200 μL of 1 M NaOH was added to the resulting mixture followed by the addition of a further 0.25 mmol of cisplatin supernatant. The mixture was left standing while being stirred at 60°C for 50 min and then for 15 days at room temperature. The volume of the filtrate was reduced to 4 mL using a vacuum concentrator consisting of Javac DD150 Double stage High Vacuum Pump Savant RVT 4104 Refrigerated Vapor Trap and Savant Speed Vac 110 Concentrator. About 20 mL of dichloromethane was added to the concentrated solution. The mixture was left standing at 5°C for 6 h. The light yellow precipitate produced was collected by filtration at the pump, washed in succession with ice cold water and ethanol. It was then air-dried. The steps in synthesis are shown in Figures 3 and 4. Attempts were also made to increase the purity of the compounds by repeated dissolution in DMF followed by precipitation with dichloromethane. However, the best results were obtained when crude products were repeatedly washed with 95% ethanol:5% water mixture.

### Characterization

#### Microanalyses

C, H, and N were determined using a Carlo Erba 1106 automatic analyzer available at the Australian National University whereas Pt was determined by graphite furnace atomic absorption spectroscopy (AAS). As QH4, QH7 and QH8, could not be obtained in crystalline form, IR, MS and ^1^H NMR spectra were used to aid in structural characterization. The IR spectra were obtained using a Varian FT-IR spectrometer (Bruker IFS66 spectrometer). To obtain mass spectra, solutions of QH4, QH7 and QH8 made in 90% methanol and 10% DMF were sprayed into a Finnigan LCQ mass spectrometer. To obtain ^1^H NMR spectra using a Bruker DPX400 spectrometer at 400.2 MHz. QH4 was dissolved in DMF, QH7 in CDCl3 and QH8 in D_2_O/DMF and prepared in 5 mm high precision Wilmad NMR tube. Spectra were referenced to internal solvent residues and all the spectra were recorded at 300 K (±1 K).

##### QH4

Yellow powder, ^1^H NMR (400 MHz, [D_6_] DMSO): δ (ppm) = 8.5 (CH ortho), 7.5 (CH meta), 4.2 (NH_3_-Pt), 3.5 (water dissolved in DMF), 2.9 (NH_2_-Pt), 2.6 (DMSO), 2.2 (CH_2_)_,_ 1.8 (CH_2_); IR (KBr): 3278.7, 3207.5, 3078.6, 2788.4, 2444.7, 1618, 1468.0, 1347.3, 1282.9, 1111.9, 1022.2, 808.0, 693.2, 501.5, 447.4 cm^−1^; MS (ESI) *m/z (%)*: 1534 (45) = [Pt_5_(3-Hydroxypridine)_5_Cl_2_(NH_3_) – 5H], 1235.11 (44) = (M + 3H), 1289 (35) = [Pt_3_(3-hydroxypyridine)_2_(NH_2_(CH_2_)_4_NH2)_3_(NH_3_)_3_Cl_2_ + 4Cl], 859.85 (53) = [Pt_2_(3-hydroxypyridine) (NH_2_(CH_2_)_4_NH2)_2_(NH_3_)Cl_3_ + 3Cl + 5H], 452.98 (100) = [Pt(3-hydroxypyridine)_2_(CH_3_NH_2_)Cl + H], 388 (64) = [Pt(NH_2_(CH_2_)_4_NH_2_) (NH_3_)_2_Cl + Cl].

Anal. calcd for C_18_H_46_Cl_6_N_10_O_2_Pt_3_: C 17.6, H 3.8, N 11.4, Cl 17.3, Pt 47.5; found: C 17.3 ± 0.4, H 3.9 ± 0.4, N 11.7 ± 0.4, Cl 17.6 ± 0.4, Pt 47.1 ± 1.0.

##### QH7

Light yellow powder, ^1^H NMR (400 MHz, [D_6_] DMSO): δ (ppm) = 8.5 (CH ortho), 8.25 (CH ortho), 7.9 (CH meta), 7.6 (CH para), 5.35 (NH), 4.4 (CH_2_-N), 3.2 (CH_2_), 2.8 (CH_2_), 1.8 ( CH_2_), δ =1.5 (CH_2_); IR (KBr): 3278.7, 3207.5, 3078.6, 2788.4, 2444.7, 1618, 1468.0, 1347.3, 1282.9, 1111.9, 1022.2, 808.0, 693.2, 501.5, 447.4 cm^−1^; MS (ESI) *m/z (%)*: 15226 (15226/2 = 763) (1.00) = [Pt_3_(3-hydroxypyridine)_5_(NH_2_(CH_2_)_6_NH_2_)_2_(NH_3_)Cl_2_ + 4Cl + 4H], 479.25 (50) = [Pt (3-hydroxypyridine)(pyridine)(NH_2_(CH_2_)_6_NH_2_) – 6H], 459.18 (30) = [Pt(3-hydroxypyridine) (NH_2_(CH_2_)_6_NH_2_)Cl_2_ – 2H].

Anal. calcd for C_17_H_52_Cl_6_N_10_OPt_3_: C 16.9, H 4.3, N 11.6, Cl 17.6, Pt 48.3; found: C 17.0 ± 0.4, H 4.6 ± 0.4, N 11.6 ± 0.4, Cl 17.5 ± 0.4, Pt 48.8 ± 1.0.

##### QH8

Greenish yellow powder, ^1^H NMR (400 MHz, [D_6_] DMSO): δ (ppm) = 8.4 (CH ortho), 7.5 (CH meta), 4.8 (NH_3_-Pt), 3.5 (water dissolved in DMF), 3.0 (NH_2_-Pt), 2.6 (CH_2_), 2.2 (CH_2_), 1.8 (CH_2_); IR (KBr): 3277.3, 3192.3, 2988.0, 2776.6, 2360.8, 1595.8, 1449.7, 1316, 1299.5, 1114.1, 1017.4, 803.8, 692.4, 553.6, 486.1, 420.7; MS (ESI) *m/z (%)*: 1276 (1276/2 = 638) (40) = [Pt_3_(3-hydroxypyridine)_4_ (NH_2_(CH_2_)_4_NH2)_2_(NH_3_)_2_Cl_3_], 675.96 (675.96/2 = 338) (90) = [Pt_2_(3-hydroxypyridine)(NH_2_(CH_2_)_4_NH_2_)Cl_2_ – 4H], The peak at m/z = 671 corresponds to [Pt_2_(NH_2_(CH_2_)_4_NH_2_) (NH_2_(CH_2_)_4_)(NH_3_)_3_Cl_2_], 430 (85) = [Pt(3-hydroxypyridine)(NH_2_(CH_2_)_4_NH_2_)(NH_3_)Cl – H], 389 (93) = Pt(NH_2_(CH_2_)_4_NH_2_)Cl_3_, 372 (97) = [Pt(NH_2_(CH_2_)_4_NH_2_)(NH_3_)Cl_2_ + H].

Anal. calcd for C_18_H_46_Cl_6_N_10_O_2_Pt_3_: C 13.5, H 3.8, N 12.1, Cl 18.4, Pt 50.7; found: C 13.8 ± 0.4, H 4.1 ± 0.4, N 11.9 ± 0.4, Cl 18.4 ± 0.4, Pt 49.9 ± 1.0.

The limiting molar conductivity values in ohm^−1^cm^2^mol^−1^ of QH2, QH3 and QH4 are respectively 336.0, 856.0 and 384.0.

#### Cytotoxicity assays

Cytotoxicity of QH4, QH7, QH8 and cisplatin (used as a reference) against human ovarian cancer cell lines, cisplatin-sensitive A2780, cisplatin-resistant A2780^cisR^ and ZD0473-resistant A2780^ZD0473R^ was determined using MTT (3-(4,5–Di-methyl-2-thiazole)-2,5-diphenyl-2H-tetrazolium bromide) reduction assay [11,12]. About 4000 to 5500 cells (maintained in logarithmic growth phase in complete medium consisting of RPMI 1640, 10% heat-inactivated fetal bovin serum (FBS), 20 mM hepes, 0.112% sodium bicarbonate, and 2 mM glutamine without antibiotics) were seeded into flat-bottomed 96-well culture plates in 10% FBS/RPMI 1640 culture medium. Platinum complexes dissolved first in a minimum volume of DMF were diluted to the required concentrations with mQ water and filtered to sterilize. Serial dilutions ranging from 0.16 μM to 20 μM in 10% FCS/RPMI 1640 medium were prepared and added to equal volumes of cell culture in quadruplicate wells. The IC_50_ values (drug concentrations required for 50% cell kill) were obtained from the results of triplicate determinations of at least three independent experiments.

#### Cellular platinum accumulation and platinum − DNA binding

The method used for the determination of cellular accumulation of platinum and the level of platinum-DNA binding was a modification of the method described by Di Blasi *et al.*[12]. In short, exponentially growing A2780, A2780^cisR^ and A2780^ZD0473R^ cells (cell density = 1 × 10^6^ cells mL^−1^) were incubated with solutions of QH4, QH7, QH8 and cisplatin (at 50 μM final concentration) for 2 h, 4 h and 24 h at the end of which cell monolayers were trypsinized and cell suspension (5 mL) was transferred to centrifuge tubes and spun at 3500 rpm for 2 min at 4°C. Ice-cold phosphate-buffered saline (PBS) was used to wash the cells twice and the pellets were stored at −20°C until assayed. At least three independent experiments were performed. For the determination of drug accumulation in the cells, cell pellets were suspended in 0.5 mL 1% triton-X, held on ice then sonicated (5 min). Total platinum contents in cell pellets were determined by graphite furnace AAS. At least three independent experiments were performed as was done for DNA binding studies.

#### Platinum − DNA binding

Following incubation of cells with drugs, high molecular weight DNA was isolated from cell pellet using JETQUICK Blood DNA Spin Kit/50 according to the modified protocol of Bowtell *et al.*[13]. DNA content was determined by UV spectrophotometry (260 nm). Pt levels were determined by graphite furnace AAS [14]. *A*_*260*_*/A*_*280*_ ratios were found to be between 1.75 and 1.8 for all samples ensuring its high purity of the DNA and the DNA concentration was calculated according to the following equation: Concentration =  Absorbance at 260 nm × 50 ng/μL.

#### Interaction with pBR322 plasmid DNA

Interaction between QH4, QH7, QH8 and cisplatin with pBR322 plasmid DNA, with and without BamH1 digestion, was studied out using gel electrophoresis based on a method described by Stellwagen [15]. The amount of DNA was kept constant while the concentrations of compounds were varied. Exactly, 1.5 μL of supplied pBR322 plasmid DNA in solution was added to solutions of the compounds at different concentrations ranging from 0.55 μM to 70 μM for QH4, QH8 and cisplatin. For QH7 concentration ranged from 0.12 μM to 15 μM. The samples were incubated for 4 h on a shaking water bath at 37°C. The reaction was stopped by rapid cooling to 0°C for 20 min. The samples were mixed with 1 μL of marker dye ethidium bromide. 16 μL of each sample was loaded onto 1% agarose gel made in TAE buffer. The gel was photographed using Eastman Kodak Company, Molecular Imaging Systems.

#### BamH1 restriction enzyme digestion

BamH1 is a type II restriction endonuclease that hydrolyses the phosphodiester bonds. It binds at the recognition sequence 5′-GGATCC-3′, and chops these sequences just after the 5′-guanine on each strand [16]. pBR322 plasmid DNA contains a single restriction site for BamH1 that converts the supercoiled form I and singly nicked circular form II to linear form III DNA. An identical set of drug-DNA mixtures as that described previously, was first incubated for 4 h on a shaking water bath at 37°C and then subjected to BamH1 (10 units μL^−1^) digestion. The mixtures were left in a shaking water bath at 37°C for another 1 h at the end of which the reaction was terminated by rapid cooling. 4 μL of ethidium bromide was added to each sample before loading onto the gel. The gel was photographed following the method described previously.

## Results and discussion

### Synthesis and characterization

The structure of QH4, Q73 and QH8 could not be confirmed by single crystal x-ray diffractometry as no suitable single crystal could be grown. However, the results of elemental analyses and spectral studies (described earlier) can be seen to provide support for the suggested structures of the compounds (Figure 1). More importantly, step-up method of synthesis starting from the central unit essentially ensures that the targeted compound results as was found to be true in the synthesis of other trinuclear platinum complexes [6-9].

### Activity against ovarian cancer cell lines

Table 1 list the IC_50_ values and resistance factors (RF) for QH4, QH7, QH8 and Cisplatin as applied to the human ovarian A2780, A2780^cisR^ and A2780^ZD0473R^ cancer cell lines. IC_50_ values are defined as drug concentrations required for 50% cell kill and RF is defined as the ratio of the concentration of drug required for 50% cell kill in the resistant cell line to that in the parent cell line.

**Table 1:** **IC**_**50 **_**(μM) values and RFs for compounds against A2780, A2780**^**cisR**^**, A2780**^**ZD0473R **^**cell lines**

**Compounds**	${\mathrm{IC}}_{50}^{\left[a\right]}$				
	**A2780**	**A2780**^**cisR**^	**RF**^**[b]**^	**A2780**^**ZD0473R**^	**RF**^**[c]**^
Cisplatin	1.09 ± 0.06	8.08 ± 1.03	7.45	4.28 ± 0.33	3.95
QH4	0.62 ± 0.11	9.24 ± 0.53	14.91	5.22 ± 0.21	8.42
QH7	8.87 ± 0.01	6.45 ± 0.47	0.73	4.61 ± 0.46	0.52
QH8	0.55 ± 0.02	4.48 ± 0.49	8.15	4.41 ± 0.22	8.02

^[a]^IC_50_ values are drug concentrations required for 50% cell death and are the means ± SD from quadruplicate determinations of at least three independent experiments. ^[b]^RF standing for resistant factor is the ratio of IC_50_ value in resistant cell line to that in the parent cell line. ^[c]^RF resistant factor applying to the cell lines A2780 and A2780^ZD0473R^.

It can be seen that QH4 and QH8 are more active than cisplatin against the parental A2780 cell line. QH8 is more active than cisplatin against the resistant A2780^cisR^ as well. Although QH7 is less active than cisplatin against all the three cell lines, it has a greater activity against the resistant A2780^cisR^ and A2780^ZD0473R^ cell lines than the parental A2780 cell line so that it has resistant factors less than one (0.73 and 0.52 for QH7 as against 8.08 and 3.95 for cisplatin). The results indicate that at the level of its activity, QH7 has been better able to overcome resistances operating in both A2780^cisR^ and A2780^ZD0473R^ cell lines. Although QH4 and QH8 have comparable activity against the parental A2780 cell line, QH4 has much lower activity than QH8 against the resistant A2780^cisR^ cell line. This difference in activity against the resistant cell line is likely to be associated with non-covalent interactions with the DNA. Whereas the central platinum ion in QH4 is bonded to two 3-hydroxypyridine ligands, it is bonded to one 3-hydroxypyridine and one ammino ligand in QH8. With the idea that in binding with DNA, QH4 would experience a greater steric hindrance than QH8, one would expect a lower level of Pt−DNA binding from interaction with QH4 than with QH8. It will be seen later that the converse is the case, possibly highlighting the role of DNA repair in cytotoxicity and the level of Pt−DNA binding.

### Cellular accumulation

**Figure 5:**
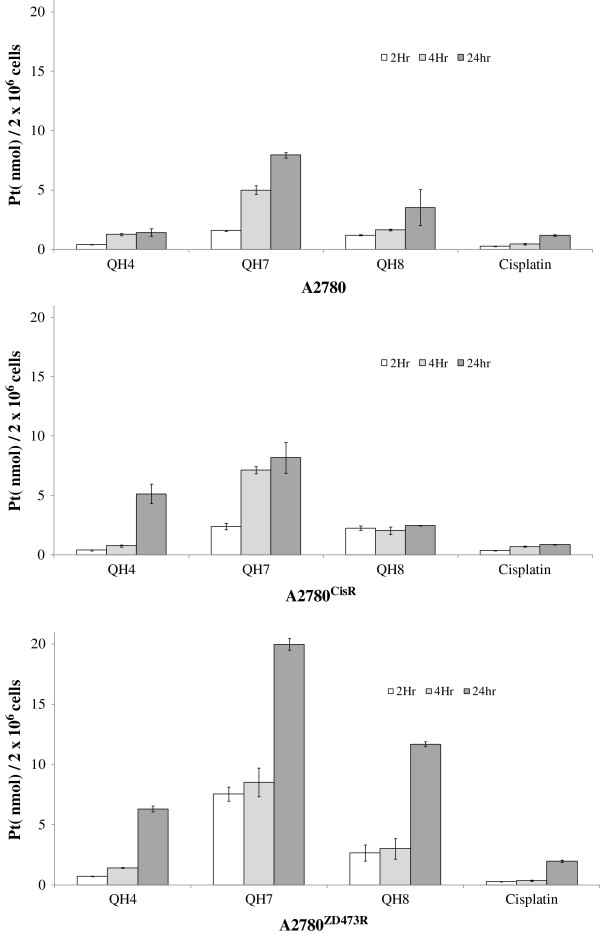
**Total intracellular platinum levels (expressed as nmol Pt per 2x10**^**6 **^**cells) found in A2780, A2780**^**cisR **^**and A2780**^**ZD0473R **^**cells after their exposure to 50 μM concentrations of QH4, QH7, QH8 and cisplatin for 2, 4 and 24 hours.**

Figure 5 gives the total intracellular platinum levels (expressed as nanomoles Pt per 2x10^6^ cells) found in A2780, A2780^cisR^ and A2780^ZD0473R^ cells after their exposure to 50 μM concentrations of QH4, QH7, QH8 and cisplatin for 2, 4 and 24 hours.

When platinum accumulations from cisplatin, QH4, QH7 and QH8 are compared, it is found that all the three trinuclear compounds QH4, QH7 and QH8 result in higher platinum accumulation than cisplatin at all time points. The results indicate that the carrier-mediated transport of QH4, QH7 and QH8 must be faster than the overall transport of cisplatin by both diffusion and the use of carriers. Surprisingly the least active compound is associated with the highest Pt accumulations in all the three cell lines at all time points, indicating that there is no clear relationship between activity and cellular accumulation of QH4, QH7 and QH8.

### DNA binding

**Figure 6:**
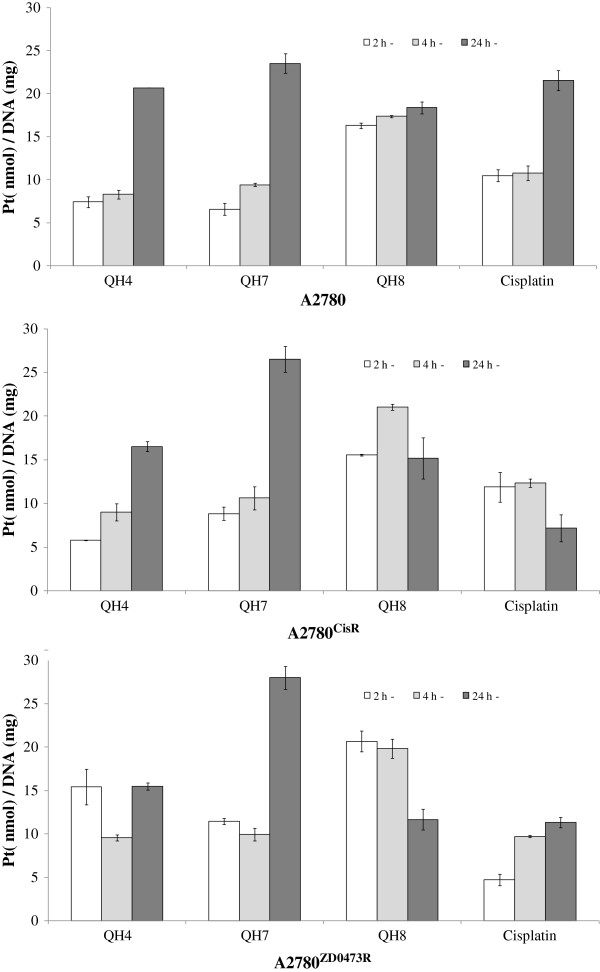
**Levels of platinum-DNA binding expressed as nmol Pt per milligram of DNA from QH2, QH7, QH8 and cisplatin in A2780, A2780**^**cisR **^**and A2780**^**ZD0473R **^**cells as a function of time.**

Figure 6 gives the levels of platinum−DNA binding expressed as nanomol of Pt per milligram of DNA from QH4, QH7, QH8 and cisplatin in A2780, A2780^cisR^ and A2780^ZD0473R^ cells as a function of time.

Among the three designed complexes, QH8 was found to be associated with the highest Pt−DNA binding levels at 2 h and 4 h but lowest at 24 h in all the three cell lines. At 2 h, QH8 had the highest level of Pt−DNA binding in A2780 cell line. The actual orders of Pt−DNA binding were:

At 2 h:

A2780 cell line: QH8 > Cisplatin > QH4 > QH7

A2780^cisR^ cell line: QH8 > Cisplatin > QH7 > QH4

A2780^ZD0473R^ cell line: QH8 > QH4 > QH7 > Cisplatin

At 4 h:

A2780 cell line: QH8 > Cisplatin > QH7 > QH4

A2780^cisR^ cell line: QH8 > Cisplatin > QH7 > QH4

A2780^ZD0473R^ cell line: QH8 > QH7 > Cisplatin > QH4

At 24 h:

A2780 cell line: QH7 > Cisplatin > QH4 > QH8

A2780^cisR^ cell line: QH7 > QH4 > QH8 > Cisplatin

A2780^ZD0473R^ cell line: QH7 > QH4 > QH8 > Cisplatin.

The results indicate that at least initially QH8 binds most readily with DNA in all the three cancer cell lines. However, whereas the binding of QH8 with nuclear DNA reaches saturation early, binding of QH4 and QH8 continues to increase such that at the end of 24 h they have overtaken QH8 in terms of Pt−DNA binding levels. The actual order of Pt−DNA binding at 24 h was:

A2780 cell line: QH7 > Cisplatin > QH4 > QH8

A2780^cisR^ cell line: QH7 > QH4 > QH8 > Cisplatin

A2780^ZD0473R^ cell line: QH7 > QH4 > QH8 > Cisplatin.

For QH8, the levels of Pt−DNA binding at 24 h in the resistant A2780^cisR^ and A2780^ZD0473R^ cell lines are actually less than the corresponding values at 4 h, suggesting that increased DNA repair must be in play as applied to the adducts formed by QH8. This is in line with the idea that increased DNA repair is a dominant mechanism of resistance operating in resistant cancer cell lines [17]. It was noted earlier that the least active compound QH7 had a greater activity against the resistant A2780^cisR^ and A2780^ZD0473R^ cell lines than the parental A2780 cell line whereas QH4 and QH8 had lower activity against the resistant cell lines than that against the parental cell line. It can be seen that for QH7 the level of Pt−DNA binding at 24 h is greatest for the A2780^ZD0473R^ cell line and least for the parental A2780 cell line. This can be seen to be in line with the order of activity of the compound. In contrast, the level of Pt−DNA binding at 24 h for QH4 is greatest in the parental A2780 cell line than the values in the resistant cell lines. As for QH8, whereas the level of Pt−DNA binding in the parental cell line continued to increase with time the values in the resistant cell lines were lowest at 24 h as compared to the values at 2 and 4 h. It is possible that DNA repair processes became more significant with time in the case of QH4 and QH8 than for QH7. It is also appropriate to note that besides having highest Pt−DNA binding levels at 24 h in all the three cell lines, QH7 was associated with highest cellular accumulation of platinum at all time points. It is suggested that the longer linker chain in QH7 (six carbons in QH7 as against four carbons in QH4 and QH8) can make it more hydrophobic so that it would bind more efficiently to hydrophobic pocket of the carrier molecule and that it was more suited to form long-range interstrand adducts with DNA based on distance match [18].

### Interaction with pBR322 plasmid DNA

**Figure 7:**
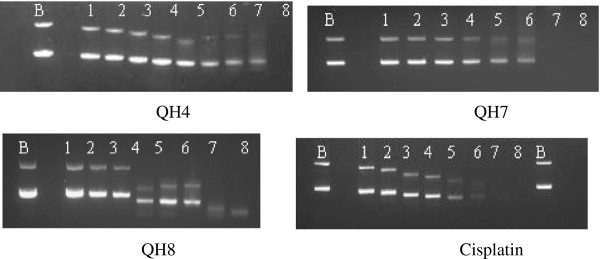
**Electrophotograms applying to the interaction of pBR322 plasmid DNA with increasing concentrations of QH4, QH7, QH8 and cisplatin.** Lane B applied to untreated pBR322 plasmid DNA to serve as a control, lanes 1 to 8 applied to plasmid DNA interacted with increasing concentrations of the compounds and cisplatin. The concentrations for QH4, QH8 and cisplatin were: lane 1: 0.55 μM, lane 2: 1.09 μM, lane 3: 2.19 μM, lane 4: 4.38 μM, lane 5: 8.75 μM, lane 6: 17.50 μM, lane 7: 35.00 μM, and lane 8: 70.00 μM. The concentrations for QH7 is as follows lane 1: 0.12 μM, lane 2 : 0.23 μM, lane 3: 0.47 μM, lane 4: 0.94 μM, lane 5:1.88 μM, lane 6: 3.75 μM, lane 7: 7.50 μM and lane 8: 15.00 μM.

When pBR322 plasmid DNA was interacted with increasing concentrations of QH4, QH7, QH8 and cisplatin, generally two DNA bands corresponding to forms I and II were observed in both untreated and treated pBR322 plasmid DNA (Figure 7).

As the concentration of the compounds was increased, the mobility of both forms I and II bands increased but at different rates such that the separation between the bands first decreased in the case of QH4, QH8 and cisplatin. The change in mobility was less significant in the case of QH7 although it was most damaging to the DNA as compared to QH4 and QH8. In the case of QH8, the separation between the bands was smallest at 4.38 μM (lane 4) above which separation was greater (lanes 5 and 6). In the case of cisplatin also, a faint coalesced band could be seen at 35.00 μM (lane 7) and no DNA band was present at 70.00 μM (lane 8). The appearance of a coalesced band followed by the presence of two distinct or nearly distinct bands at higher concentration indicates the change in the conformation of the form I DNA from being negatively supercoiled form I though relaxed circular form I to positively super coiled form I [18]. A single but faint coalesced band observed at still higher concentrations of QH8 (7.50 μM and 15.00 μM applying to lanes 7 and 8) possibly is indicative of DNA damage rather than any change in DNA conformation. The most active compound QH4 can be seen to be slightly more damaging to DNA than QH8 but less than QH7.

### BamH1 digestion

**Figure 8:**
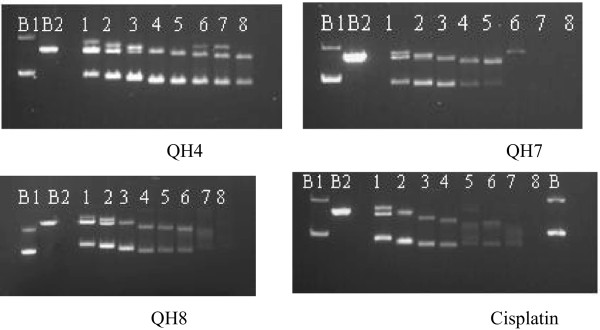
**Electrophoretograms applying to the incubated mixtures of pBR322 plasmid DNA and varying concentrations of QH4, QH7, QH8 and cisplatin followed by digestion with BamH1.** Lane B_1_ applied to the untreated pBR322 plasmid DNA and undigested with BamH1, lane B_2_ applied to untreated but digestion with BamH1. Lanes 1 to 8: apply to pBR322 plasmid DNA interacted with increasing concentrations of the compounds followed by BamH1 digestion. The concentrations for QH4, QH8 and cisplatin were: lane 1: 0.55 μM, lane 2: 1.09 μM, lane 3: 2.19 μM, lane 4: 4.38 μM, lane 5: 8.75 μM, lane 6: 17.50 μM, lane 7: 35.00 μM, and lane 8: 70.00 μM. The concentrations for QH7 is as follows lane 1: 0.12, lane 2 : 0.23, lane 3: 0.47, lane 4: 0.94, lane 5:1.88, lane 6: 3.75, lane 7: 7.50 and lane 8: 15.00.

To gain further insight into changes in DNA confirmation, drug-DNA incubation as above was followed by BamH1 digestion. Figure 8 gave the electrophoretograms applying to the interaction of pBR322 plasmid DNA with increasing concentrations of QH4, QH7, QH8 and cisplatin for a period of 4 h at 37°C followed by BamH1 digestion for a further period of 1 h at the same temperature.

When pBR322 plasmid band was digested with BamH1 (B_2_), only one band corresponding to form III band was observed whereas in the untreated and undigested pBR322 plasmid DNA (B_1_). In the case of QH4, three bands corresponding to forms I, II and III were observed in lanes 1, 2 and 3 applying to concentrations 0.55 μM, 1.09 μM and 2.19 μM respectively. Two bands were observed in lanes 4, 5 and 8 applying to concentrations 4.38 μM, 8.75 μM and 70.00 μM respectively. The appearance of three bands in lanes 6 and 7 corresponding to concentrations 17.50 μM and 35.00 μM respectively, is possibly an artifact of the presence of excess enzyme. In the case of QH7, three bands corresponding to forms I, II and III were observed in lane 1 corresponding to the concentration 0.12 μM. Two bands corresponding to forms I and II were observed in lanes 2 to 5 for concentrations ranging from 1.09 μM and 8.75 μM and a single faint could be seen in lane 6 corresponding to concentration 17.50 μM. No band can be seen in lanes 7 and 8. In the case of QH8, three bands corresponding to forms I, II and III were observed in lanes 1 and 2, and only forms I and II bands were observed in lanes 3 to 8. The results indicate that among the designed complexes, QH7 is most able to induce changes in DNA conformation and to cause most DNA damage even though it is less active, in fact the least active compound against the parent cell line A2780. The lower activity of QH7 but a greater ability to cause DNA damage and induce changes in DNA conformation can be seen to highlight complex nature of the relationship between drug−DNA binding and activity. It may be noted that whereas binding of platinum drug with DNA brings about changes in DNA conformation and can induce DNA damage, cell death through apoptosis is actually carried out by downstream processes in the cell cycle in which many proteins are involved. In the case QH8, three bands corresponding to forms I, II and III were observed in lanes 1 and 2 corresponding to the concentrations 0.55 μM and 1.09 μM respectively, two bands corresponding to forms I and II were observed in lanes 3 to 6 for concentrations ranging from 2.19 μM to 17.50 μM and a much fainter band could be seen in lane 7 corresponding to concentration 35.00 μM. No band can be seen in lane 8. In the case of cisplatin, three bands corresponding to forms I, II and III were observed in lane 1 corresponding to the concentration 0.55 μM and two bands corresponding to forms I and II were observed in lanes 2 to 7 corresponding to concentrations ranging from 1.09 μM, to 35.00 μM. No band can be seen in lane 8. Table 2 gives provides a list of the bands.

**Table 2:** Bands observed after BamH1 digestion of pBR322 plasmid DNA interacted with increasing concentrations of compounds

**Compounds**	**Bands observed in lanes 1 to 8**							
	**1**	**2**	**3**	**4**	**5**	**6**	**7**	**8**
QH4	I, II, III	I, II, III	I, II, III	I, II	I, II	I, II, III	I, II, III	I, II
QH7	I, II, III	I, II, III	I, II	I, II	I, II	I, II	I,II	No band
QH8	I, II, III	I, II, III	I, II	I, II	I, II	I, II	I,II	No band
Cisplatin	I, II, III	I, II	I, II	I, II	I, II	I, II	I,II	No band

The numbers I, II and III stand respectively for supercoiled form I DNA, singly nicked circular form II DNA and linear form III DNA.

## Conclusion

Three triplatinum complexes with *cis*-geometry for terminal metal centres and coded as QH4, QH7 and QH8 have been synthesized and investigated for activity against ovarian tumour models. QH8 has been found to be more active than cisplatin against parental and resistant human ovarian tumour models.

## Abbreviations

QH4: [{*cis*-PtCl(NH_3_)_2_}_2_ μ{*trans*-Pt(3-hydroxypyridine)_2_(H_2_N(CH_2_)_4_NH_2_)_2_}]Cl_4_; QH7: [{*cis*-PtCl(NH_3_)_2_}_2_ μ{*trans*-Pt(3-hydroxypyridine)(NH_3_)(H_2_N(CH_2_)_6_NH_2_)_2_}]Cl_4_; QH8: [{*cis*-PtCl(NH_3_)_2_}_2_ μ{*trans*-Pt(3-hydroxypyridine)(NH_3_)(H_2_N(CH_2_)_4_NH_2_)_2_}]Cl_4_; DH6Cl: [{*trans*-PtCl(NH_3_)_2_}_2_ μ{*trans*-Pt(NH_3_)_2_(H_2_N(CH_2_)_6_NH_2_)_2_}]Cl_4_; YH11: [*trans*-PtCl_2_(NH_3_)(3-hydroxypyridine)]; CH1: [*trans*-PtCl_2_(3-hydroxypyridine)_2_; MTT: 3-[4,5-dimethylthiazol-2yl]-diphenyl tetrazolium bromide; DMF: N,N-dimethylformamide; DMSO: Dimethyl sulfoxide (DMSO).

## Competing interests

The authors declare that they have no financial interest and personal relationships with other people or organizations that could in appropriately influence (bias) their work.

## Authors’ contributions

SAH developed the methodology, acquired and interpreted data, and drafted the manuscript. PB and JQY aided in study design. FH conceived and designed the study, developed the methodology, interpreted data, edited the manuscript, and oversaw the study. All authors have read and approved the final manuscript.
